# Toward Unifying Global Hotspots of Wild and Domesticated Biodiversity

**DOI:** 10.3390/plants9091128

**Published:** 2020-08-31

**Authors:** Samuel Pironon, James S. Borrell, Ian Ondo, Ruben Douglas, Charlotte Phillips, Colin K. Khoury, Michael B. Kantar, Nathan Fumia, Marybel Soto Gomez, Juan Viruel, Rafael Govaerts, Félix Forest, Alexandre Antonelli

**Affiliations:** 1 Royal Botanic Gardens, Kew, Richmond TW93AQ, UK; j.borrell@kew.org (J.S.B.); i.ondo@kew.org (I.O.); r.douglas@kew.org (R.D.); j.viruel@kew.org (J.V.); r.govaerts@kew.org (R.G.); f.forest@kew.org (F.F.); a.antonelli@kew.org (A.A.); 2 Royal Botanic Gardens, Kew, Wakehurst Place TW93AE, UK; c.phillips@kew.org; 3 International Center for Tropical Agriculture (CIAT), Cali 6713, Colombia; c.khoury@cgiar.org; 4 Department of Biology, Saint Louis University, St. Louis, MO 63103, USA; 5 Department of Tropical Plant and Soil Science, University of Hawaii at Manoa, Honolulu, HI 96822, USA; mbkantar@hawaii.edu (M.B.K.); nfumia@hawaii.edu (N.F.); 6 Department of Botany, University of British Columbia, Vancouver, BC V6T1Z4, Canada; marybel.soto@gmail.com; 7 UBC Botanical Garden and Centre for Plant Research, University of British Columbia, Vancouver, BC V6T1Z4, Canada; 8 Gothenburg Global Biodiversity Centre, Department of Biological and Environmental Sciences, University of Gothenburg, 40530 Göteborg, Sweden

**Keywords:** agro-biodiversity, breeding, centres of origin, conservation, crop wild relatives, domestication, geographic distribution, phylogenetic diversity, useful plants, Vavilov centres

## Abstract

Global biodiversity hotspots are areas containing high levels of species richness, endemism and threat. Similarly, regions of agriculturally relevant diversity have been identified where many domesticated plants and animals originated, and co-occurred with their wild ancestors and relatives. The agro-biodiversity in these regions has, likewise, often been considered threatened. Biodiversity and agro-biodiversity hotspots partly overlap, but their geographic intricacies have rarely been investigated together. Here we review the history of these two concepts and explore their geographic relationship by analysing global distribution and human use data for all plants, and for major crops and associated wild relatives. We highlight a geographic continuum between agro-biodiversity hotspots that contain high richness in species that are intensively used and well known by humanity (i.e., major crops and most viewed species on Wikipedia) and biodiversity hotspots encompassing species that are less heavily used and documented (i.e., crop wild relatives and species lacking information on Wikipedia). Our contribution highlights the key considerations needed for further developing a unifying concept of agro-biodiversity hotspots that encompasses multiple facets of diversity (including genetic and phylogenetic) and the linkage with overall biodiversity. This integration will ultimately enhance our understanding of the geography of human-plant interactions and help guide the preservation of nature and its contributions to people.

## 1. Introduction

Biogeographers and conservation biologists have long been interested in identifying and characterizing geographic regions containing a higher concentration of biodiversity and derived natural resources than surrounding areas, ranging from within- and among-species diversity through to ecosystem services [1,2,3], at different spatial, temporal and taxonomic scales [4]. Centres (also known as hotspots) and peripheries (coldspots) of plant diversity have been shown to be unevenly distributed and to play a fundamental role in shaping ecosystems and delivering associated benefits to humans and other species [5,6]. Mapping efforts contribute to a better fundamental understanding of both biodiversity (e.g., species extinction, diversification and co-existence) [7], and the interaction between people and nature, including the resulting socio-economic benefits and threats [8,9,10,11]. This is a particularly urgent endeavour in the current context of a rapidly growing global human population with increasing consumer expectations, posing a serious threat for both plant diversity and its long-term contributions to people [12]. Factors, such as land use and climate change, pollution, direct exploitation of species, and biological invasions have direct and indirect impacts on plant diversity [13,14], potentially undermining current and unrealised plant-based adaptive solutions [15,16] and traditional knowledge associated with plant uses [17]. Documenting the distribution of plant diversity and its uses are, therefore, critical steps towards developing the transformative changes required to achieve socio-economic sustainability, while preserving life on Earth.

Conservationists, constrained by finite resources, have used the concept of biodiversity hotspots to advocate for the allocation of international efforts and resources in regions of the world containing exceptionally diverse, unique and threatened biodiversity [18]. However, biodiversity hotspots often fail to capture the multi-faceted nature of biodiversity. For example, hotspot designations may consider a narrow range of organisms [19], and miss non-terrestrial habitats [3], phylogenetic and functional diversity (but see [20]). The seminal definition of a biodiversity hotspot, which was based solely on plants from tropical forests, highlights this shortcoming [21]. An additional limitation of biodiversity hotspots, as currently defined, is that they rarely consider anthropogenic interactions as anything other than a threat [22]. However, the sustainable and responsible use of nature, as demonstrated by traditional small-scale livelihoods and indigenous communities around the world, presents opportunities for resolving the current biodiversity crisis and global challenges facing humanity [12,15,23]. Finally, although often considered implicitly [13,18], nature’s contributions to people are rarely considered when setting global conservation priorities, except for a few regulating ecosystem services [2,3,24].

Agro-biodiversity is a sub-component of biodiversity that accounts for the variety of life that contributes to food and agriculture [25]. In the broadest sense, it can be broken down into two components: (a) Planned agro-biodiversity, which refers to the diversity within and across species (domesticated or undomesticated) that are used by people, and (b) associated agro-biodiversity, which refers to species that surround and/or enhance planned agro-biodiversity [26]. In order to identify species and forms of high potential for improving and sustaining agriculture, research efforts have generally focused on mapping centres of origin and diversity of major domesticated plant species (mostly food crops) and associated wild relatives [27,28]. While the distribution of plants of highest importance for commodity production and human nutrition is now widely studied [6,29], much uncertainty remains about the large fraction of neglected and underutilized species that contribute to a wide array of provisioning, support, regulation and cultural services [30]. Moreover, although recent international treaties incentivized the preservation of plant genetic resources in the face of major global challenges [30], the distribution of useful plant genetic diversity is mainly studied through proxies (e.g., taxonomy, geographic distribution and environmental data) [28]. Likewise, the drivers and spatial patterns of decline in agro-biodiversity remain poorly understood [15,17].

Here we explore the relationship between hotspots of wild plant diversity and regions containing high levels of agro-biodiversity by (i) reviewing the history of the two concepts, (ii) characterizing the degree to which they overlap in space and the human and biological drivers of these geographic (in-) congruencies, and (iii) considering how to better integrate the multi-faceted aspects of useful plant diversity, including genetic and phylogenetic diversity. Finally, we propose a new general framework and discuss future avenues for obtaining an improved understanding and preservation of global hotspots of useful plant diversity [31,32].

## 2. The History of Diversity Hotspots

### 2.1. Biodiversity Hotspots

**Figure 1:**
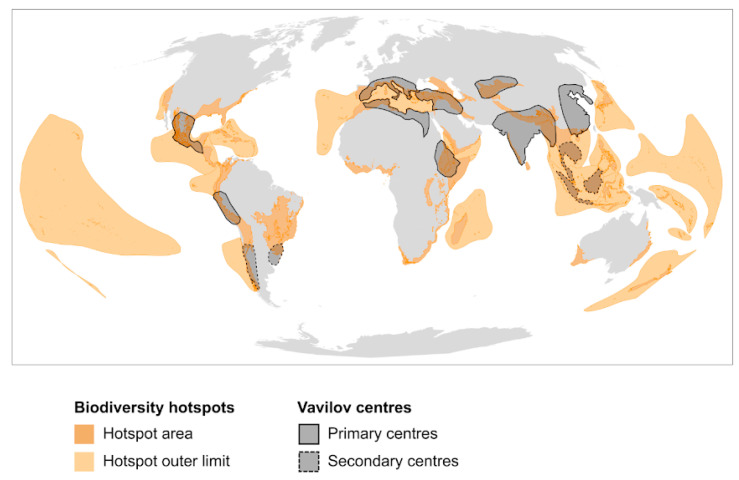
Global distribution of biodiversity hotspots and Vavilov centres. Biodiversity hotspots were first defined by Myers in 1988 [21], and now comprise 35 regions of high species richness, endemism and threat, as last updated in 2011 by Mittermeier and colleagues [18]. Islands constituting biodiversity hotspots are highlighted by outer hotspot limits. Vavilov first defined centres of origin of cultivated species and wild relatives in 1924; he provided an update in 1935, comprising eight primary and three secondary centres [27].

The distribution of life on Earth has long been investigated by naturalists aiming to understand, preserve and exploit the natural world [33,34,35]. These influential observations were followed by a more systematic documentation of the potential distribution of plant diversity globally based on the compilation of data from regional checklists and pioneering modelling efforts [36,37,38]. It was only in 1988 that British environmentalist Norman Myers (1934–2019) coined the term “hotspot” to define 10 tropical forest areas considered to be both irreplaceable (i.e., due to high concentrations of endemic plant species) and vulnerable (i.e., due to high rates of deforestation) [21]. Eight hotspots were added subsequently, including four in Mediterranean regions [39]. Later, the definition of a hotspot underwent a major update by considering strict quantitative criteria to designate areas containing at least 0.5% of the world’s flora, but less than 30% of its original vegetation—resulting in the addition of seven additional hotspots [40]. A recent major update incorporated new data and took account of both terrestrial vertebrates and plants to delineate a total of 35 global biodiversity hotspots (Figure 1) [18]. Besides their importance for biodiversity, hotspots are home to more than two billion people and encompass some of humanity’s highest population growth, as well as poverty rates [22].

Since the definition of the hotspot concept by Myers, several other approaches have been explored to define and refine important biodiversity areas. Alongside terrestrial plants and vertebrates, other taxa have been considered to date, including marine mammals [41], phytoplankton [42] and soil invertebrates [43]. Conservationists have also argued for the protection of remaining wilderness, considered to be the most ecologically intact areas of the world, as these have been shown to experience substantially fewer threats, and thus, contribute more to the persistence of biodiversity [44]. Whereas, early work focused on species richness, recent studies have also advocated for the consideration of functional traits and evolutionary history, which may permit a better understanding of nature’s contribution to both people and nature itself [45]. Incorporating knowledge across various disciplines is of particular importance as the multiple facets of biodiversity that they represent often do not overlap in space [20,46,47]. Spatial congruencies between biodiversity and direct measures of ecosystem services, such as carbon and water, are also increasingly being investigated [2,3].

Plants have been central to the definition of biodiversity hotspots since the pioneering work of Myers. Regions of high plant diversity have been investigated in multiple ways in the last decades, mainly by attempting to obtain finer continuous maps of (rare) plant species richness, as opposed to considering categorical hotspots. Barthlott and colleagues produced an influential estimate at the scale of ecoregions [5,48,49]. In contrast, researchers at the Royal Botanic Gardens, Kew gathered information at a semi-administrative scale [50,51], and adapted and expanded the concept of Important Plant Areas to the tropics [52], a programme that remains under development [53]. More recently, vascular plant species richness has been interpolated globally based on ~1000 local estimates [54], extrapolated based on the relationship between the occurrences of ~200,000 species and their environment [3], or investigated through the examination of commonness-rarity patterns [55]. Hotspots of high plant richness and endemism include Mesoamerica, the tropical Andes, the Amazon, Brazil’s Atlantic rainforest, Central Africa, the western Ghats, South-East Asia, and many islands (e.g., Madagascar, New Guinea), mountainous regions (e.g., Alps, Caucasus) and Mediterranean areas (e.g., Cape floristic region). Despite their international recognition, most of these regions have become increasingly depleted under ongoing human pressures, whereas richness may increase at their periphery through repeated introductions in gardens and disturbed habitats [56]. These processes are leading to a global loss of diversity and increasing biotic homogenisation [57]. In this context, mapping priority areas and taxa for conservation remain, at least, as crucial and urgent as when hotspots were initially identified more than three decades ago.

### 2.2. Agro-Biodiversity Hotspots

Starting some 150 years ago, global botanical, geographic, linguistic, and archaeological evidence were combined to identify the geographic origins of crops, including distinctions among Old versus New World species [58]. These works were largely built on developments in plant systematics (e.g., Linnaeus (1707–1787), Alefeld (1820–1872), de Candolle (1806–1893)), phytogeography (e.g., Willdenow (1765–1812), von Humboldt (1769–1859), Wegener (1880–1930)), and evolution by natural and artificial selection (e.g., Darwin (1809–1882)). A number of these scientists were extensive travellers, whose contributions to their fields were catalysed by their voyages. However, none travelled as much as Nikolaï I. Vavilov (1887–1943). Informed by previous phytogeographic research and the rediscovery of Gregor J. Mendel’s primary works in genetics, the Russian agricultural scientist pursued genetic variation in crops and their wild relatives, exploring five continents over several decades. Through his field experiences, Vavilov came to propose a set of independent “centres of origin” of cultivated food plants around the world, based fundamentally on where he saw a maximum concentration of diversity of traditional varieties of a wide range of crops, along with their wild relatives. Vavilov initially proposed three centres of origin of forms (1924), progressing to as many as eight primary centres, and including several sub-centres (Figure 1). These putative centres, which later in his tragically curtailed life he called hearths of origin, included Mesoamerica; parts of the Andes, Chile and Brazil-Paraguay; the Mediterranean; the Near East; Ethiopia; Central Asia; India; China; and Indo-Malaysia [27].

Since Vavilov, the regions of origin and diversity of different crops have been debated, investigated and refined, benefiting from an expanding body of archaeological, linguistic, genetic, and taxonomic information [6,59,60,61,62,63,64,65,66]. “Centres of diversity” came to be the preferred term over “centres of origin”, to account for the difficulty in assigning an exact place of origin for most crops, and due to the understanding that high concentrations of crop varieties and related wild species are not in every case located where crops were initially domesticated [62]. “Regions” (also “megacentres” per Zhukovsky and “non-centres” per Harlan) rather than “centres” became preferred to reflect the large size of many of these geographic areas, and again, the difficulty of pinpointing exact locations where crops were domesticated [6,67]. At the present time, multidisciplinary evidence supports the identification of ca. 24–28 different areas around the world where crop domestication occurred independently, mostly beginning in the early to middle Holocene (approximately 11,700–6000 years ago), and in a few cases more recently [66,68,69]. Not all of the identified areas would be considered by most researchers as a “centre” or “region” of origin or diversity, as only a limited number of crops were domesticated in some of these.

There was an acceleration in the movement of crop plants across the globe between 1500–1700, as they were introduced to colonizing countries, their colonies and other regions with emerging export-oriented production [9,70]. Agricultural development and globalisation have made a number of crop species available to consumers worldwide, but in turn, increased homogeneity in global agriculture [71,72]. Added to the geographic decoupling of agricultural production and consumption [73,74], this homogenisation has deteriorated the connection between crops and their geographic origin [6]. Nevertheless, these areas of origin continue to hold foremost importance with regard to crop genetic diversity as their crops diversified for thousands of years under natural and human selection, including via further introgression with wild relatives [75,76]. During Vavilov’s voyages a century ago, it was already apparent that the diversity of crops that people grew and consumed was changing as a result of globalisation. Major efforts commenced, particularly during the 1970s and 1980s, to collect traditional crop landraces and wild relatives for safeguarding in genebanks, and to also support in situ conservation, often in collaboration with subsistence agriculturalists [77]. Such efforts continue today, often linked to seed banking and germplasm collections, based on the recognition of persisting gaps in conservation of agro-biodiversity [28,78].

The history of agro-biodiversity was primarily written by colonial powers and white male explorers, conferring little or no room to traditional knowledge holders [79]. Moving ahead in tackling the challenges of mapping, understanding, protecting and further exploring the potential of crop plants and associated wild relatives, it is crucial that benefits of this work are shared in equitable ways [80]. In particular, access must be ensured in low-income countries to new or neglected crops, especially those that offer climate resilience, nutritious contents and other desirable traits.

## 3. From Biodiversity to Agro-Biodiversity Hotspots: A Geographic Continuum

The geographic distribution of biodiversity and agro-diversity hotspots has long been investigated, but rarely together. Very little is known about their spatial intricacies despite recent calls for their integration [31]. Several areas of high plant diversity do not include primary regions of agro-biodiversity (e.g., California, Caribbean, Brazilian Cerrado, South Africa, Madagascar, Pacific islands) and a few agro-biodiversity regions are not recognized as diversity hotspots (e.g., large parts of Eastern Asia and India) (Figure 1). Although human activities are altering this pattern [12,14], global plant species richness generally decreases with increasing latitude [5,54], reflecting past environmental changes, land configuration and the evolutionary histories of species [81,82]. On the other hand, the history of global human migrations, civilisations, economy and cultural preferences have been profoundly intertwined with the distribution and availability of natural resources (species richness, abundance and properties) to shape regions of agro-biodiversity [9]. Here, we present a new set of analyses to explore, illustrate and discuss the spatial congruence between biodiversity and agro-biodiversity hotspots, and its relationship with two important processes—human selection of species and species evolutionary history.

### 3.1. From Popularity to Anonymity

Vavilov provided an early spatial representation of the origins of cultivated plants by mapping the distribution of major food species. However, by restricting (justifiably) his focus mainly to selected food plants encountered in his field experiences, Vavilov was unable to produce a comprehensive assessment of the distribution of all plant species selected for use by humans across the world. Identifying which plant species are most selected by humans for a broader range of uses in addition to food, and characterizing their geographic distribution is, thus, key for defining agro-biodiversity hotspots and for relating their variation to the wider context of biodiversity.

In our currently globalized world and big data era, it is now possible to investigate human preferences for species more extensively. Here we assessed the popularity of most vascular plants on the free online encyclopaedia Wikipedia (www.wikipedia.org), using search data as a proxy for cultural preference, knowledge of plant species, use by humans and domestication intensity. As Wikipedia can be edited by anyone at any given time, it cannot be considered a reliable source of information without critical evaluation. However, our analysis remains independent from the quality of Wikipedia articles as it only examines the interaction between users and the web platform by quantifying numbers of page views. Species names and occupied geographic regions were retrieved for 339,924 species from the World Checklist of Vascular Plants (WCVP) [51]. Wikipedia uses the taxonomic backbone of the WCVP, so no additional name matching was performed. Geographic regions were retrieved at the national or sub-national level (finest level three) of the World Geographical Scheme for Recording Plant Distribution (WGSRPD), which was developed by the International Working Group on Taxonomic Databases for Plant Sciences [83]. We retrieved species popularity on Wikipedia as measured by the number of page views over the period 1 January 2016 to 1 January 2020 using the R package pageviews. This information relates to English Wikipedia pages only as it is the language with the highest number of pages overall and often the source of translations into other languages. We ranked these data according to three categories: (1) The 1000 most popular species; this includes plants used as food, medicine, timber, ornamentals and cosmetics (Supplementary Materials), (2) the remaining species covered by Wikipedia (i.e., those with an available page), (3) the remaining species not documented in Wikipedia (i.e., those without a page).

**Figure 2:**
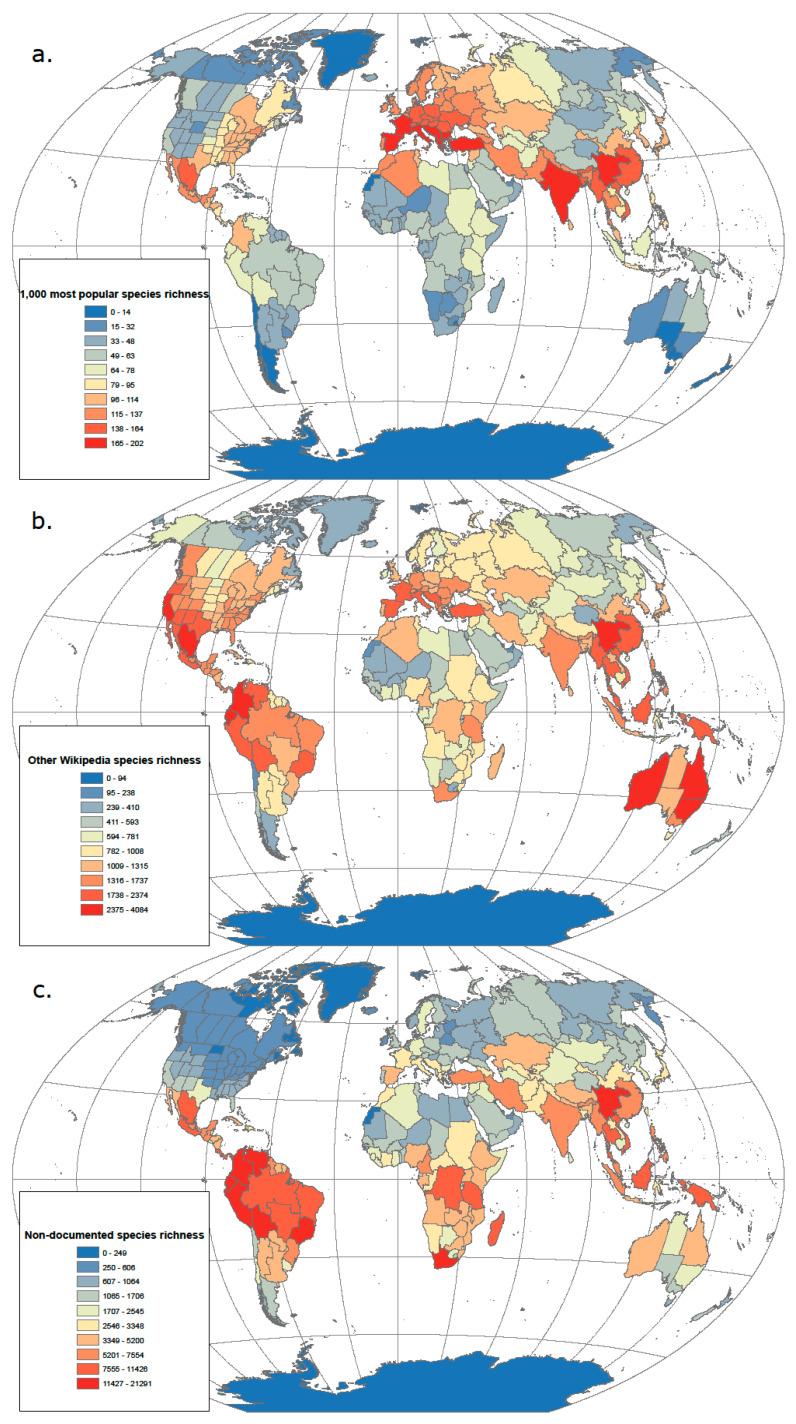
Global distribution of the species richness of plants ranging from popular to anonymous based on English Wikipedia page views. Global distribution of (**a**) the 1000 most popular plant species on Wikipedia; (**b**) 49,019 species documented in Wikipedia, excluding the 1000 most popular ones; (**c**) 280,905 species not documented in Wikipedia. Popularity was measured as the number of views of the Wikipedia webpage of each species. Native distribution data was retrieved from the World Checklist of Vascular Plants at the national or sub-national level of the World Geographical Scheme for Recording Plant Distribution (WGSRPD) [51].

The global richness distribution of the 1000 most popular species is strikingly similar to the Vavilov primary centres with particularly high richness in subtropical regions of the Northern hemisphere (Figure 2a). There are also expansions towards temperate areas (i.e., Eastern North America, Europe and Central Asia), while the Northern Andes, Eastern Africa and the Indo-Malayan regions have relatively low crop richness at a global scale, but high richness within their respective continents. Richness generally increases towards the tropics for species that are documented in Wikipedia but fall outside of the 1000 most popular category, with particularly high concentrations in the Mediterranean and subtropical regions that are characterized by high plant species endemism but low richness in major crops (e.g., Western North America, South Africa, Australia) [5] (Figure 2b). In contrast, richness in species not documented in Wikipedia tends to follow a latitudinal gradient similar to that observed for total plant diversity and most similar to biodiversity hotspots (Figure 2c) [18,84]. These findings highlight the relationship between the spatial structure of biodiversity and agro-biodiversity, and people’s knowledge, perception and use of nature. Indeed, we observe the existence of a geographic continuum between popular plant diversity that may be more intensively used by humanity (based on the existence of a Wikipedia page; 2A) and anonymous plant diversity that may be less heavily used (based on the lack of a Wikipedia page; 2C). One artefactual limitation in our assessment of the distribution of plant popularity is the general over-representation of English-speaking regions (e.g., United States, Canada, Australia) given that our data extraction came from English Wikipedia pages only. This also likely explains relatively low values of popular species richness in regions, such as the Andes or Ethiopia (Figure 2a), although those are still visible at the continental scale.

### 3.2. From Domesticates to Wild Relatives

Alongside major crops, Vavilov was also interested in documenting, mapping, collecting, using, and preserving wild ancestors and closely related species [27]. By considering less known and undomesticated (or less domesticated) parent and sister species, Vavilov directly connected his definition of regions of origin/diversity with wild plant diversity. Recent studies illustrate this link between biodiversity and agro-biodiversity hotspots; these assessed the distribution of the closest and more distant relatives of major crops and found high species richness in plant diversity hotspots, such as the Brazilian Cerrado and Atlantic Forest or South-East Asia [28,85].

Here we assessed the current distribution of 222 major international crops and 2,731 of their wild relative species using comprehensive lists from the USDA ARS GRIN-Global Taxonomy and geographic data from the World Checklist of Vascular Plants, again retrieved at the national and sub-national level (level three) of the WGSRPD [51]. Crop wild relatives are classified across three gene pools based on both their relatedness (using phylogenetics and systematics) and crossing ability with the crop [86,87]: gene pool one comprises the most closely related (even conspecific) wild species that are generally fully interfertile with the crop; gene pool two includes more distant relatives that may be crossed to the crop with more difficulty; and gene pool three typically contains the most distantly related and least compatible species within the genus to which the crop belongs (sometimes including other genera). Here, we assess changes in geographic patterns across a gradient from cultivated species to their closest wild relatives to their more distant wild relatives, by mapping richness for crops and each associated gene pool separately. When more than one species was identified in a gene pool for a given crop, we merged their distribution to assign the same weight to each crop, thus, avoiding overrepresentation of genera with many wild relatives. Geographic data were not available at the infra-specific level (i.e., sub-species, varieties, forms) for all taxa, and so we performed analyses at the species level.

**Figure 3:**
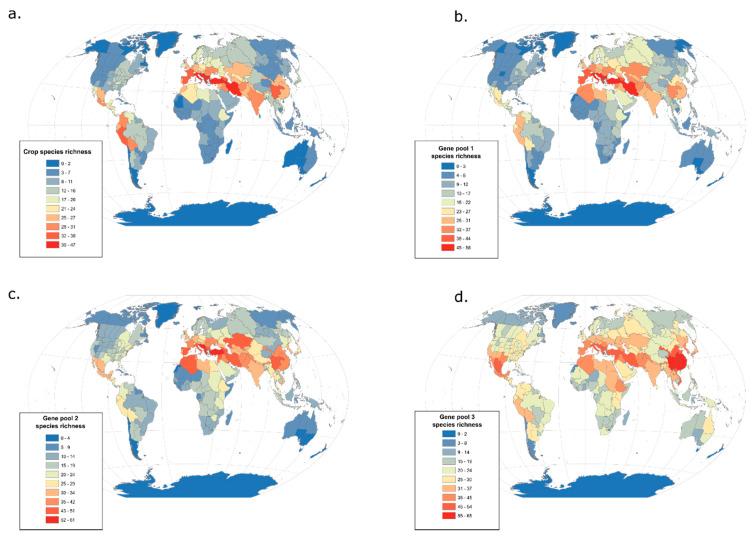
Global distribution of the richness of major crops and their wild relatives. Global distribution of (**a**) 222 major crops, (**b**) 361 wild relative species in gene pool one, (**c**) 1040 wild relative species in gene pool two and (**d**) 2358 wild relative species in gene pool three; see text for gene pool characterisation. Species identities and gene pool classifications were retrieved from the USDA ARS GRIN Global Taxonomy. Distribution data was retrieved from the World Checklist of Vascular Plants at level three of the World Geographical Scheme for Recording Plant Distribution (WGSRPD) [51]. When more than one wild relative species was identified for a crop in a gene pool, we merged their distribution to assign the same weight to each crop and avoid genera with many wild relatives to be overrepresented.

Geographic patterns in major crop species richness strongly overlap with the Vavilov centres (Figure 3a) and are also similar to the 1000 most popular plant species on Wikipedia (Figure 2a). Given that gene pool one is composed of the closest crop wild relatives, including progenitors and/or wild types of the crop species, the distribution of gene pool one species richness is very similar to that of the crops (Figure 3b). Slight increases and decreases are respectively observed inside and outside Europe, which may be explained by more extensive documentation of European crop wild relatives compared to those that occur in other regions [88]. Gene pools two and three provide a more diffuse representation of the Vavilov centres: species richness decreases in most of the Vavilov centres, but increases in surrounding regions, particularly (but not exclusively) towards the tropics and plant diversity hotspots (Figure 3c,d). Although centres of crop diversity remain visible when mapping secondary and tertiary gene pools, the geographic signal diminishes as we move further away from the most explored and popular branches of the tree of life. In contrast, areas of high biodiversity start to emerge, which is even more striking when species richness mapping does not account for the over-representation of genera with many wild relatives [28,85]. This reinforces the existence of a geographic continuum between agro-biodiversity (i.e., widely used and cultivated crops) and biodiversity (i.e., non-domesticated and less used sister species of crops) related to the strength of the interaction between humans and plants, but also plant evolutionary history.

## 4. Integrating Genetic and Phylogenetic Diversity into Agro-Biodiversity Hotspots

The designation of hotspots of plant species richness and rarity has provided a way to focus attention on the intrinsic value of biodiversity at a global scale. However, the contributions of plants to livelihoods are rarely considered in this context, despite the predominance of utilitarian arguments in conservation. Going beyond species counts (i.e., taxonomic diversity) to describe and understand structural and/or chemical properties of species and their diversity is of critical importance. The wide range of plant properties cannot currently be quantified across all species but is often related to genetic variation within and among species [89]. Characterizing genetic and phylogenetic diversity and their geographic distribution may, therefore, provide a useful framework for identifying other currently unknown forms of diversity and associated usages, and for preserving plant genetic resources.

### 4.1. Hotspots of Phylogenetic Diversity

Some species are the sole remaining representative of ancient lineages, while others are part of recent and rapid radiations (i.e., increases in species richness related to elevated speciation rate) that may comprise hundreds of closely related species. Therefore, conservation biologists now account for the fact that the evolutionary histories of species are not equivalent when setting priorities [90]. To address this inconsistency, phylogenetic diversity (PD) was proposed as an approach based on evolutionary information [91]. The PD of a given area is equal to the sum of all the branches on a phylogenetic tree linking the set of species that occur in this area. Areas containing high PD will, therefore, reflect higher concentrations in distantly related species. While previous studies have cast doubt on the ability of PD to provide a different answer to species richness for prioritisation [92,93], the decoupling of biodiversity patterns, based on taxon richness and evolutionary history, have since been clearly demonstrated (e.g., Reference [94]). Phylogenetic diversity and associated metrics have been widely used to explore biodiversity patterns among and within biodiversity hotspots [95,96,97].

**Figure 4:**
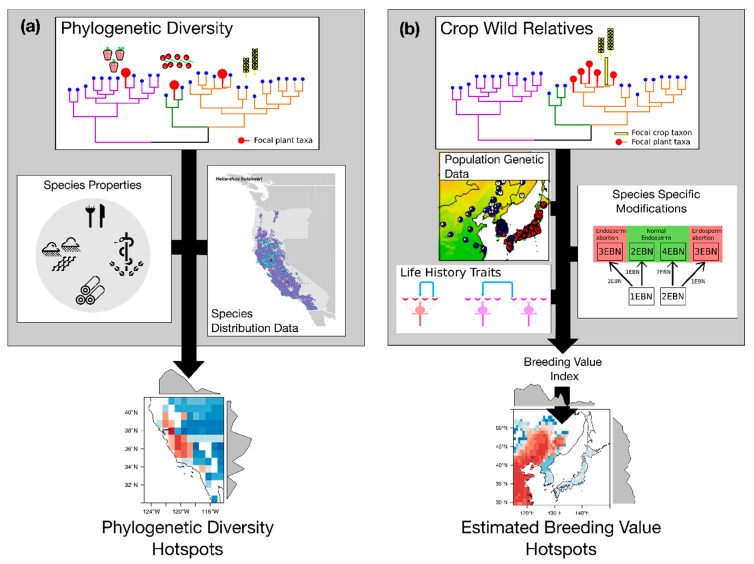
A proposed general framework for the further inclusion of genetic information into the mapping of agro-biodiversity hotspots. (**a**) A relationship between species evolutionary history and their physical or chemical properties (i.e., features or phenotype) would use phylogenetic diversity as a proxy for feature diversity, the latter being less readily quantifiable across wide ranges of species, regions and features. Combined with species distribution data, phylogenetic diversity could ultimately identifies hotspots of feature diversity and priority areas for the conservation of species’ contribution to people; (**b**) By considering phylogenetic information together with population genomics, cytology, life history and/or ecological data, an Estimated Breeding Value (EBV) could be computed for crop wild relative species and/or populations. Combined with distribution data, hotspots of EBV could then be mapped to identify areas containing high concentrations in valuable wild gene sources for preservation and crop improvement.

One of the most important characteristics of PD is its potential to act as a surrogate for feature diversity (i.e., the diversity of characters or traits of species), which encompasses the qualities of plants that are beneficial for humans. While the relationship between PD and feature diversity remains contentious [45,98], it is nevertheless an attribute that is of particular importance for the identification of areas rich in crop wild relatives and species with unexplored uses for humans. Assuming that PD is a suitable surrogate for feature diversity, maintaining PD would not only help retain the evolutionary potential of species, but also maximise the potential unanticipated benefits that biodiversity may have in the future for humans (i.e., biodiversity option values), particularly in the face of global change [99,100]. In the context of agro-biodiversity hotspots and the identification of new sources of plant properties, PD as a metric of choice has a key role to play (Figure 4a). Ultimately, however, the identification of hotspots of agro-biodiversity would be best served by the integration of various metrics capturing the multiple facets of biodiversity [101], and also by considering the human dimension for better understanding the portion, intensity and modes of use of biodiversity by humanity.

### 4.2. Hotspots of Breeding Value

Genetic diversity represents the raw material that humans have relied upon for millennia for the maintenance and improvement of crops. Plant breeding is a long-term process [102] aimed at enhancing traits of interest (e.g., yield, quality, disease tolerance, abiotic stress tolerance) using extant variation [103]. There is tremendous genetic and phenotypic diversity in crop wild relatives distributed across the plant tree of life [69,104]. Identifying the top priority branches (from species to populations) that will generate the largest changes in trait values, while having the closest form to current crops is of great interest to plant breeders and agriculturalists. Populations that exist at the edges of distribution ranges may have great utility for breeding as these often occur in more isolated and extreme ecological conditions and display high levels of genetic and phenotypic differentiation [1,66,78,105]. Identification of potentially useful crop wild relatives is generally based on heuristic approaches (e.g., place X has a similar environment to place Y, so translocation is expected to lead to positive results), or by using large-scale germplasm collections to search for specific traits of interest [106,107]. However, these approaches, as currently applied, may not fully explore available plant genetic diversity (e.g., Reference [108]) and are often not available for crops that are less economically important.

There have been recent efforts to identify the geographic regions where crop wild relative species are concentrated [28]. Characterisation of the distribution of populations that are most compatible with existing crops and exhibit phenotypes of interest has somewhat lagged behind. Nevertheless, there are now concerted efforts to identify and incorporate these taxa into breeding programmes for producing viable new cultivars by using integrative approaches that leverage large amounts of data from phylogenetics, population genomics, cytology, life history, ecological niches, and predictions of future environmental conditions that crops may experience [78,109,110,111].

Building on historic advancements, it may be possible to incorporate the data currently used in heuristic approaches for crop wild relative identification into a more general framework to help the decision-making within breeding programmes. Such a framework could potentially be modelled from the Estimated Breeding Value (EBV), a common breeding programme metric that could expedite selection of donor species. An EBV is the potential of an individual as a genetic parent, considering the heritability of a given trait under selection [103]. Typically, EBVs are obtained from narrow breeding populations of a single species by multiplying the narrow-sense heritability (calculated either by variance decomposition or parent-offspring regression) by the difference between the parent performance and population mean, which provides an estimate of how the progeny of a specific parent will perform relative to an average parent. We propose that EBVs could also be calculated for crop wild relative species and populations, as long as the phenotype of interest is clearly defined. Applied to species and populations instead of individuals, the EBV could go beyond heritability to additionally incorporate biological factors (e.g., ploidy, mating system), evolutionary factors (e.g., phylogenetic relationship), and ecological factors (e.g., species environmental niche) in a hierarchical way for prioritizing species that may be of greater potential use to plant breeding. Functionally, this proposed use of EBV would produce a ranking of species for an individual breeding programme, based on the desired phenotype by crossing interaction. Ultimately, characterizing this variation in utility across species (and populations) may help identify priority areas for in- and ex-situ conservation related to specific breeding targets (Figure 4b).

## 5. Towards a Unified Concept of Agro-Biodiversity Hotspot

**Figure 5:**
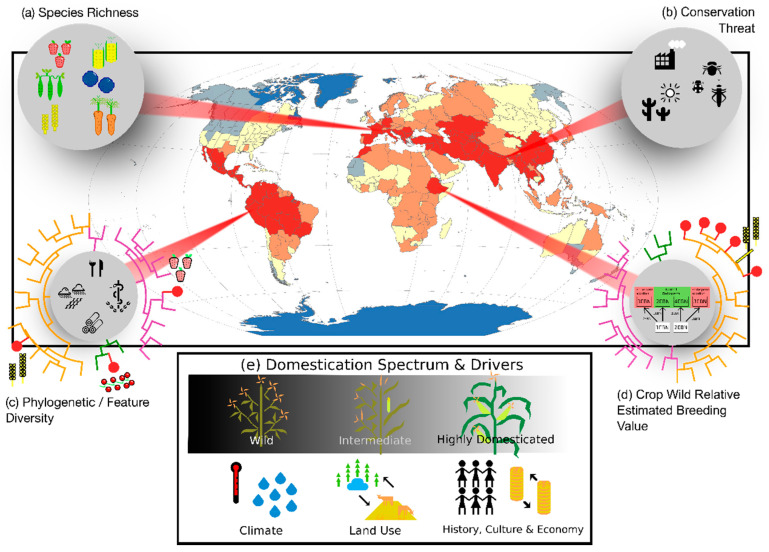
The conceptual framework for the identification of agro-biodiversity hotspots, including (**a**) plant species richness (applicable to infra-specific levels as well); (**b**) threats; (**c**) species (or infra-specific taxa) evolutionary and features diversity; (**d**) crop wild relatives estimated breeding value; and accounting for (**e**) the geographic continuum between hotspots of wild species diversity and regions containing high concentrations in major crops (i.e., highly domesticated species), and its environmental and human drivers. The map does not provide a new estimate of the distribution of agro-biodiversity hotspots, but rather illustrates a combination of the potential two ends of the domestication spectrum: Major crop species richness (Figure 3a) and species richness undocumented in Wikipedia (Figure 2c). High to low species richness is represented from red to blue.

Hotspots of both biodiversity and agro-biodiversity have long relied on counting numbers of species (i.e., species richness; Figure 5a) and assessing threats (Figure 5b). While biodiversity scientists have mainly focused on numbers of rare species (including many narrowly distributed taxa) for conservation, agronomists have been interested in diversity within gene pools (i.e., numbers of domesticated species and wild relatives) [27,28,85] and within crops (e.g., numbers of landraces) [112]. Although taxon counts remain extremely useful, new approaches are now proposed to account for the multi-faceted nature of (agro-)biodiversity, such as functional diversity accounting for the diversity in species chemical properties and eco-/agri-system functions [113], phylogenetic diversity as a potential proxy for functional and property diversity (Figure 5c) or for identifying gene sources for breeding programmes (Figure 5d). As highlighted by their seminal definition from N. Myers and recent publications [114], biodiversity hotspots are also deeply related to the distribution of a wide range of threats, many of which are shared with agro-biodiversity (e.g., land use and climate change, pollution, biological invasions, over-harvesting), but less formally included in the geographic assessments of the latter (Figure 5b) [12,16]. Although the different facets and threats of agro-biodiversity are not all expected to overlap geographically, our paper proposes to assess them jointly rather than separately.

Primary regions of agro-biodiversity have focused on relatively few important crops selected by researchers, whereas they have mainly explored wild relatives of these domesticated species, which are not always considered for other uses than crop improvement. Given the existence of a wide domestication spectrum, ranging from major global crops (i.e., highly domesticated species) to those harvested in the wild [16], we believe that further work on regions of agro-biodiversity should expand the focus to better include the long list of plants that provide food and other cultural benefits to humanity (Figure 5e). This will be made effective through the documentation of the tremendous diversity of neglected and under-utilized species of the world (with more than 30,000 useful plant species known to date [115]). Many of these species occur naturally in low-income countries, including already established biodiversity hotspots, which are also often home to large human populations and cultural diversity [22,116]. Understanding the drivers of the distributions of nature’s contribution to people across the domestication spectrum (from climate and land use to socio-economic factors; Figure 5e) is also fundamental to define hotspots and design conservation and development efforts to sustain socio-environmental sustainability.

The recognition of biodiversity hotspots and agro-biodiversity (Vavilov) centres have played important roles to raise public awareness, foster research and attract political action to preserve and use natural resources sustainably. Given the urgency and magnitude of the global challenges outlined by the United Nations’ Sustainable Development Goals and the recent report on biodiversity loss by the intergovernmental science-policy platform on biodiversity and ecosystem Services (IPBES) [12], it is more important than ever to refine, integrate and disseminate such powerful concepts. Failing to protect hotspots of natural resources, and especially agro-biodiversity, would have damaging consequences on nature and human livelihoods, both at those centres and in their peripheries. Our paper calls for the further development and integration of a range of commonly used and more recently proposed indices, while accounting for the key interaction with biodiversity, into the agro-biodiversity hotspot concept.

## Supplementary Materials

The following are available online at https://www.mdpi.com/2223-7747/9/9/1128/s1.
